# Tocotrienol-Rich Fraction Ameliorates the Aluminium Chloride-Induced Neurovascular Dysfunction-Associated Vascular Dementia in Rats

**DOI:** 10.3390/ph16060828

**Published:** 2023-06-01

**Authors:** Sohrab A. Shaikh, Arunachalam Muthuraman

**Affiliations:** Pharmacology Unit, Faculty of Pharmacy, AIMST University, Semeling, Bedong 08100, Kedah, Malaysia

**Keywords:** endothelial dysfunction, myeloperoxidase, platelet-derived growth factor-C, serum nitrite, tocotrienol-rich fraction, vascular dementia

## Abstract

Neurovascular dysfunction leads to the second most common type of dementia, i.e., vascular dementia (VaD). Toxic metals, such as aluminium, increase the risk of neurovascular dysfunction-associated VaD. Hence, we hypothesized that a natural antioxidant derived from palm oil, i.e., tocotrienol-rich fraction (TRF), can attenuate the aluminium chloride (AlCl_3_)-induced VaD in rats. Rats were induced with AlCl_3_ (150 mg/kg) intraperitoneally for seven days followed by TRF treatment for twenty-one days. The elevated plus maze test was performed for memory assessment. Serum nitrite and plasma myeloperoxidase (MPO) levels were measured as biomarkers for endothelial dysfunction and small vessel disease determination. Thiobarbituric acid reactive substance (TBARS) was determined as brain oxidative stress marker. Platelet-derived growth factor-C (PDGF-C) expression in the hippocampus was identified using immunohistochemistry for detecting the neovascularisation process. AlCl_3_ showed a significant decrease in memory and serum nitrite levels, while MPO and TBARS levels were increased; moreover, PDGF-C was not expressed in the hippocampus. However, TRF treatment significantly improved memory, increased serum nitrite, decreased MPO and TBARS, and expressed PDGF-C in hippocampus. Thus, the results imply that TRF reduces brain oxidative stress, improves endothelial function, facilitates hippocampus PDGF-C expression for neovascularisation process, protects neurons, and improves memory in neurovascular dysfunction-associated VaD rats.

## 1. Introduction

Dementia is a long-term, progressive condition that impairs cognitive function in a way that is not related to natural ageing [1]. Neurovascular dysfunction is associated with an increasing list of pathologies that includes vascular dementia [2]. Vascular dementia (VaD) is the second most prevalent type of dementia, and the incidence of an overlapping syndrome of Alzheimer’s disease and VaD is higher than initially thought. Furthermore, developing nations have a higher prevalence [3]. There are currently no approved standard treatments for VaD because the underlying cause of the disease is not fully understood [4]. In cerebrovascular diseases, such as VaD, the endothelium is essential. After a stroke, endothelial dysfunction (ED) develops, which damages the blood-brain barrier and causes oxidative stress, inflammation, and increased vascular tone [5]. Moreover, damage to the endothelium and the neurovascular unit (NVU) appears to be a major factor in the pathogenesis of vascular cognitive impairment [6] or VaD. It is crucial to know that cerebral small vessel disease (SVD), the leading cause of vascular dementia, triples the risk of stroke [7] and that the NVU plays a major role in the pathophysiological process of ischemic stroke [8]. The known pathophysiological changes in VaD have led to the suggestion of different pharmacological treatment options [4]. However, there is a gap in understanding the management of VaD caused due to risk factors, such as heavy metals and its treatment approach.

Heavy metal exposure is associated with SVD because it can cause oxidative stress, which interferes with the nitric oxide signalling system and eventually results in ED [9]. Importantly, ED is also considered a key in the pathogenesis of SVD and VaD [10]. Aluminium is one of the harmful metals that are frequently utilised in daily life. It enters the body easily from several sources, mainly through drinking water and eating food [11]. Additionally, it is well-known that aluminium is neurotoxic and that exposure to it is linked to the development of neurodegenerative diseases, such as dementia. This association may be explained by the buildup of aluminium in vulnerable brain regions, such as the hippocampus and frontal cortex [12]. Hence, exposure to aluminium increases the likelihood of developing VaD, possibly by increasing the oxidative stress in the brain, which may then result in the development of SVD and eventually lead to ischemic stroke and ED, impairing the functioning of NVU.

Targeting the NVU should lead to new insights for potent therapeutic approaches and make it easier to translate experimental results into clinical setup [13]. Medications that target the NVU can prevent ischemic stroke [8], and using antioxidants in particular may have the desired effect by lowering brain oxidative stress and improving endothelial function. In this context, numerous studies on stroke emphasise how drugs or naturally occurring plant-derived substances (curcumin, carnosic acid, and epicatechin) can prevent stroke [13]. Furthermore, individuals with VaD exhibit perfusional changes and neovascularisation; nevertheless, studies show that neoangiogenesis is not effectively able to occur in VaD patients due to their narrower vessels [6,14]. Therefore, to induce neovascularisation, activating PDGF-C with exogenous substances may be a novel strategy in VaD management.

In this situation, using a natural compound with known antioxidant activity would be a good choice to halt the advancement of VaD brought on by aluminium. One of the naturally occurring antioxidants is palm oil tocotrienol-rich fraction (TRF) which is a mixture of tocotrienols and tocopherols extracted from palm plant fruit (*Elaeis guineensis*) [15]. Several pharmacological effects of TRF, including antioxidant [16,17], anti-inflammatory, and neuroprotective, have been documented [18]. A rising body of clinical trial data also supports that palm oil tocotrienol may aid in the prevention of dementia and Alzheimer’s disease [19]. Recently, we have reported the TRF effectiveness in managing VaD in type 2 diabetic rats [20]. However, TRF effects in reducing neurovascular ED brought on by aluminium chloride (AlCl_3_)-induced VaD in rats is not yet reported. Biomarkers, such as serum nitrite for ED and plasma myeloperoxidase for SVD, can help identify cerebrovascular functioning; moreover, identifying PDGF-C expression in the hippocampus may help in understanding the neovascularisation process. Hence, this study focuses on neurovascular endothelial dysfunction-associated VaD in rats and its treatment approach. Therefore, based on the facts, we hypothesized that TRF treatment may attenuate the AlCl_3_-induced neurovascular dysfunction-associated VaD in rats, perhaps by reducing oxidative stress, SVD, ED, and facilitating the neovascularisation process by activating PDGF-C.

## 2. Results

### 2.1. Effect of TRF on Learning and Memory Function during the Elevated Plus Maze (EPM) Test

**Figure 1:**
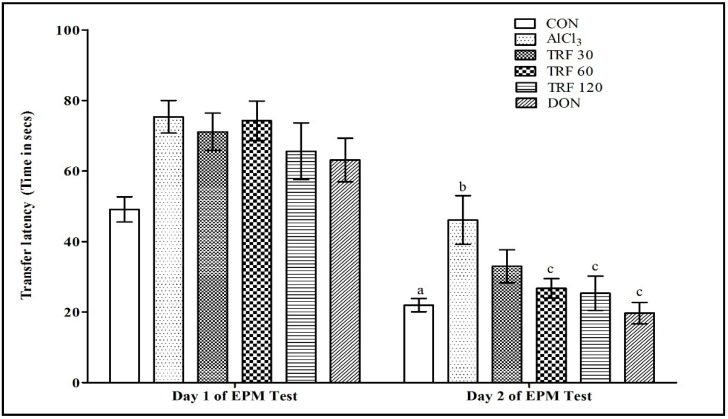
Effect of TRF on transfer latency (TL) time in an elevated plus maze test. a = *p* < 0.05 vs. day 1 TL of CON; b = *p* < 0.05 vs. day 2 TL of CON; c = *p* < 0.05 vs. day 2 TL of AlCl_3_. Each group (*n* = 8) represents mean ± SEM. Abbreviations: AlCl_3_, aluminium chloride; CON, control; DON, donepezil; EPM, elevated plus maze; and TRF, tocotrienol-rich fraction.

There was a highly significant (*p* < 0.01) decrease in day 2 transfer latency (TL) compared to day 1 TL in the normal control (CON) group rats, indicating normal learning ability. Further, there was a highly significant (*p* < 0.01) increase in day 2 TL in the AlCl_3_ group rats compared to day 2 TL of the CON group rats, indicating AlCl_3_-induced impairment of memory. However, there was a significant (*p* < 0.05) and (*p* < 0.01) decrease in day 2 TL in the TRF 60 and TRF 120 group rats, respectively, compared to day 2 TL of the AlCl_3_ group rats. Similarly, there was a highly significant (*p* < 0.001) decrease in day 2 TL found in the donepezil (DON) group rats compared to day 2 TL of the AlCl_3_ group rats, hence, indicating that TRF and DON mitigate AlCl_3_-induced impairment of memory (Figure 1).

### 2.2. Effect of TRF on Brain-Thiobarbituric Acid Reactive Substances (TBARS) Levels

There was a highly significant (*p* < 0.01) increase in TBARS levels found in the AlCl_3_ group rats compared to the CON group rats, indicating AlCl_3_-induced increased brain oxidative stress. However, there was a highly significant (*p* < 0.01) decrease in TBARS levels found in the TRF 60, TRF 120, and DON group of rats compared to the AlCl_3_ group rats; thus, indicating that TRF and DON reduced AlCl_3_-induced brain oxidative stress level (Table 1).

### 2.3. Effect of TRF on Serum Nitrite Levels

**Figure 2:**
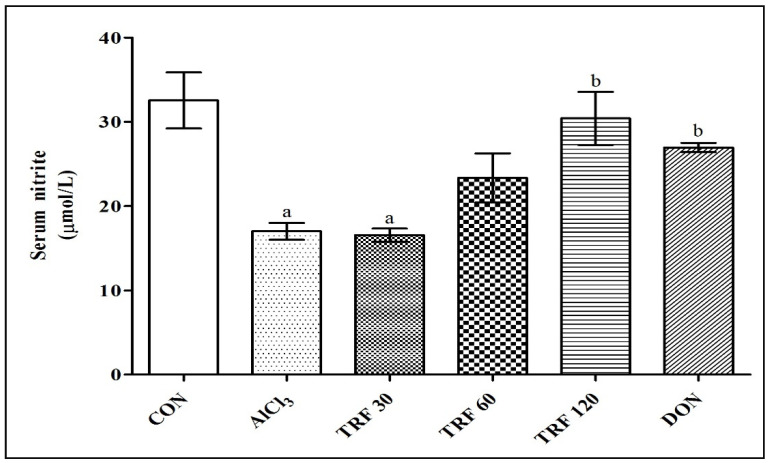
Effect of TRF on serum nitrite levels. a = *p* < 0.05 vs. CON; b = *p* < 0.05 vs. AlCl_3_. Each group (*n* = 6) represents mean ± SEM. Abbreviations: AlCl_3_, aluminium chloride; CON, control; DON, donepezil; and TRF, tocotrienol-rich fraction.

There was a highly significant (*p* < 0.001) decrease in serum nitrite levels found in the AlCl_3_ and TRF 30 group rats compared to the CON group rats, indicating AlCl_3_-induced endothelial dysfunction. However, there was a highly significant (*p* < 0.01) and significant (*p* < 0.05) increase in the serum nitrite levels found in the TRF 120 and DON group rats respectively compared to the AlCl_3_ group rats; thus, indicating that TRF and DON reduced AlCl_3_-induced endothelial dysfunction (Figure 2).

### 2.4. Effect of TRF on Plasma Myeloperoxidase (MPO) Levels

**Figure 3:**
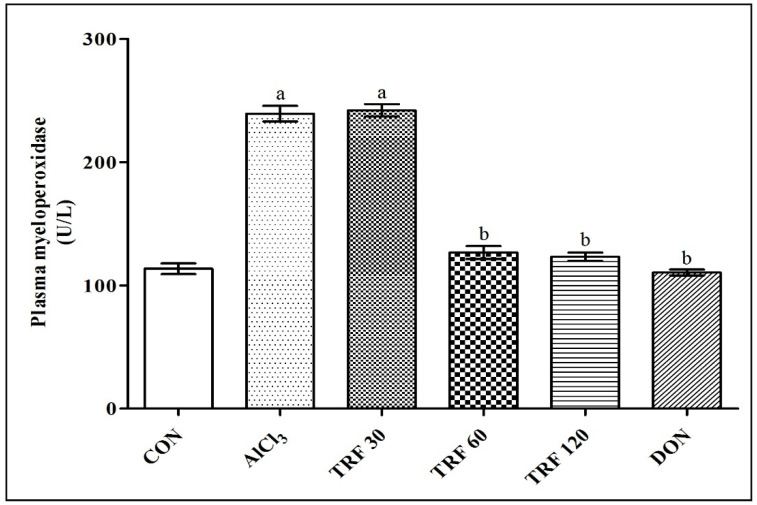
Effect of TRF on plasma myeloperoxidase levels. a = *p* < 0.05 vs. CON; b = *p* < 0.05 vs. AlCl_3_. Each group (*n* = 6) represents mean ± SEM. Abbreviations: AlCl_3_, aluminium chloride; CON, control; DON, donepezil; and TRF, tocotrienol-rich fraction.

There was a highly significant (*p* < 0.001) increase in plasma MPO levels found in the AlCl_3_ and TRF 30 group rats compared to the CON group rats indicating AlCl_3_-induced vascular inflammation and SVD development in rats. However, there was a highly significant (*p* < 0.001) decrease in plasma MPO levels found in the TRF 60, TRF 120, and DON group of rats compared to the AlCl_3_ group rats; thus, indicating that TRF and DON may have reduced AlCl_3_-induced vascular inflammation and SVD development in rats (Figure 3).

### 2.5. Effect of TRF on Hippocampus PDGF-C Expression

**Figure 4:**
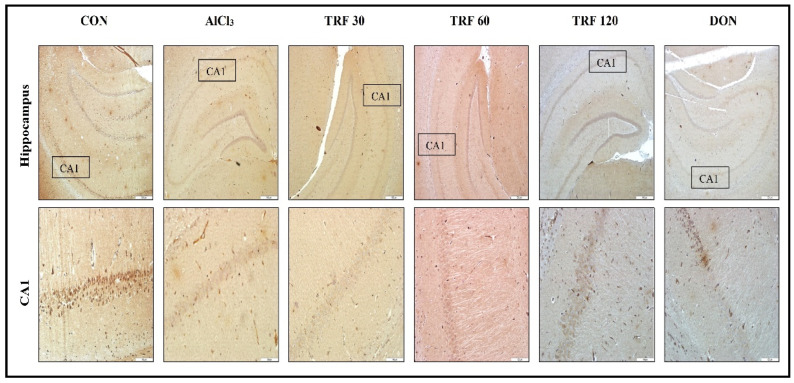
Microscopic images representing immunohistochemical staining of PDGF-C expression in the hippocampus (upper panel 40× magnification; scale bar 500 µm) and its corresponding CA1 area (lower panel 200× magnification; scale bar 100 µm). The high-magnification images in the lower panel indicate PDGF-C expression in the CA1 area. The PDGF-C expression is strongly positive in the CON group rats, faintly positive in the AlCl_3_ and TRF 30 group rats, mid-positive in the TRF 60 and TRF 120 group rats, and strongly positive in the DON group rats. Abbreviations: AlCl_3_, aluminium chloride; CA1, cornu ammonis 1; CON, control; DON, donepezil; PDGF-C, platelet-derived growth factor-C; and TRF, tocotrienol-rich fraction.

The PDGF-C expression in the hippocampus was confirmed by observing immunostained coronal brain sections under the microscope and rating the staining intensity. The CON group rat showed strong positive immunostaining in the cornu ammonis 1 (CA1) area of the hippocampus; thus, indicating the presence of PDGF-C expression. In contrast, the AlCl_3_ and TRF 30 group rats showed faint positive immunostaining; hence, indicating the decreased or absence of PDGF-C expression, perhaps due to AlCl_3_-induced neurovascular toxicity. Notably, the TRF 60 and TRF 120 group rats showed mid-positive immunostaining while the DON group rats showed strong positive immunostaining in the CA1 area; thus, indicating the presence of PDGF-C expression perhaps for the neovascularisation process. These immunohistochemistry findings, therefore, put forward TRF and DON possible roles in stimulating PDGF-C at the neurovascular site to reduce AlCl_3_-induced neurovascular changes (Figure 4).

## 3. Discussion

Acute administration of AlCl_3_ to rats in the current investigation was effective in causing vascular dementia, which is related to endothelial dysfunction [21]; this may be because aluminium can remain in the body for a longer time to exert its neurotoxic effects [22,23]. Moreover, the hippocampus is particularly susceptible to the toxic effects of aluminium, which could cause neurodegeneration and eventually memory loss or dementia. The EPM test was used in this work to evaluate memory function, and it was found that AlCl_3_ rats showed considerable memory loss. The results are similar with previously published studies on memory loss caused by aluminium [24,25]. In contrast, TRF therapy prevented memory loss in AlCl_3_ rats. TRF, because of its antioxidant ability, may have improved memory in VaD rats by lowering brain oxidative stress and preventing neuronal death in the hippocampus caused due to aluminium toxicity. Moreover, these results are consistent with other research [26,27,28] that demonstrates the influence of TRF on memory protection. 

Further, this study reveals TRF’s noteworthy antioxidant activity. The oxidative destruction of lipids is a result of lipid peroxidation. The two main byproducts are hydroperoxides and lipid peroxyl radicals. It has been shown that these oxidised lipids lead to inflammation and endothelial dysfunction [29]. Cerebrovascular disorders are brought on by unbalanced lipid peroxidation [30]. Aluminium is a pro-oxidant [31] that can promote lipid peroxidation and oxidative stress by producing free radicals. AlCl_3_ has also been found to disrupt metabolism, particularly for low-molecular-weight antioxidants, such as glutathione, which accelerates the rate of lipid peroxidation in the brain [32]. Brain lipid peroxidation in this study was determined by measuring TBARS levels [33]. We found elevated TBARS levels in AlCl_3_ rats, demonstrating increased brain oxidative stress. Our findings are also supported by previously published reports [34,35]. Nevertheless, TRF administration in AlCl_3_ rats decreased TBARS levels, indicating its antioxidant action. Moreover, our findings are consistent with earlier studies with TRF [36,37,38,39,40,41]. It is suggested that chelation therapy may be an effective strategy to reduce the toxic metal load and the associated complications [9]; hence, the possible mechanism of TRF could be not only by disrupting free radical chain reactions [37] but also by chelating aluminium (prooxidant metal) and neutralizing free and lipid peroxy radicals [42]. We further presume that TRF would have engaged the nuclear factor erythroid 2-related factor 2 (Nrf2) pathway in our study to provide antioxidant activity because it has been documented that TRF activates the Nrf2 pathway [16,17]. Furthermore, according to research, superoxide anion produced by reactive oxygen species (ROS) quickly reacts with nitric oxide (NO) to form peroxynitrite, which reduces NO bioavailability and causes endothelial dysfunction [9]. Atherosclerosis and other macro- and microvascular disorders are caused by a dysfunctional endothelium, which is essential for the initiation of vascular dysfunction [2]. In the current investigation, we discovered considerably lower serum nitrite levels in AlCl_3_-induced rats, which indicate endothelial dysfunction. Similar decreases in serum nitrite levels have been associated with exposure to heavy metals [9,43] and these findings are also in line with other studies that revealed lower serum nitrite levels in VaD [44,45]. Conversely, rats treated with TRF increased serum nitrite in AlCl_3_ rats perhaps by reducing the oxidative stress that reverses the nitric oxide synthase activity, and this findings is in line with previous studies with TRF [46]. TRF action on the vascular system is further supported by the fact that palm oil is rich in tocotrienols, a type of vitamin E [19], and more importantly, vitamin E was found to increase nitric oxide production, which in turn serves to improve the endothelial-dependent vasodilatation by relaxing the vascular smooth muscle cells, is further evidence of TRF action on the vascular system [18]. Moreover, earlier studies with compounds such as lithium suggest that increased nitric oxide production improves the endothelial-dependent vasodilatation and may be important in cerebrovascular disease [47,48].This finding suggests that TRF therapy reduced neurovascular dysfunction, which may have enhanced vascular function in NVU and safeguarded neurons and memory in VaD rats.

ROS are also important contributors to SVD. It is reported that MPO levels were significantly found to be elevated in patients with cerebral SVD [49]. In the present study, we found elevated plasma MPO in AlCl_3_-induced rats; thus, indicating the sign of vascular damage, perhaps cerebrovascular damage, since, MPO has the unique capacity to oxidise calcium ions utilising hydrogen peroxide and consequently induce vascular injury [50], it is possible that cerebrovascular damage would result. Additionally, MPO has been demonstrated to exhibit pro-atherogenic and pro-oxidant capabilities, suggesting that it may act as a marker and mediator of vascular inflammation [51,52]. However, treatment with TRF was able to attenuate the raised plasma MPO in AlCl_3_-induced VaD rats, indicating reduction in proatherogenic markers. Moreover, our findings are in accordance with previous studies suggesting that TRF eliminated excessive myeloperoxidase activation, which is in line with the TRF plasma antioxidant activity [53,54]. Thereby, this finding may suggest that TRF therapy halted the vascular inflammation process possibly the cerebral SVD development and improved the neurovascular functioning in VaD rats.

The key finding of this research was the immunohistochemistry-based identification of PDGF-C expression in the hippocampus. According to reports, aluminium may play a role in the pathophysiology of stroke [23] and may result in an ischemic stroke in the cerebral small vessels. Furthermore, one-fourth of ischemic strokes are caused by cerebral small vessel disease, and it is a major factor in the development of vascular dementia [49]. In humans, PDGF-C is typically expressed in the majority of adult organs and tissues, including the brain, and is necessary for the proper development of the vascular network [55]. According to studies, after an ischemic insult, PDGF-C increases angiogenesis and revascularises ischemic tissues [56]. Furthermore, it appears that PDGF-C-based investigations are undertaken in particular to promote its neuroprotection, proliferation, angiogenesis, and anti-apoptotic characteristics [57]. In this investigation, we found reduced PDGF-C expression in the hippocampus of AlCl_3_-induced VaD rats, which may indicate either reduced or no neovascularisation process in the damaged region of the hippocampus. Moreover, according to earlier studies, PDGF-C expression is downregulated in ischemic tissues, which impairs angiogenic activity [56]. However, AlCl_3_-induced VaD rats treated with TRF exhibited significant PDGF-C expression, suggesting that TRF may have promoted the endogenous PDGF-C, a neuroprotective factor, to protect neurons from AlCl_3_-induced apoptosis. Moreover, it is possible that TRF-mediated PDGF-C activation would have provided the antioxidant effect, since, it has recently been reported that PDGF-C diminishes the oxidative stress brought on by high glucose levels by increasing superoxide dismutase 2 (SOD2) expression and SOD activity and modulating the Keap1 expression gene [58]. Furthermore, we correlate our PDGF-C findings with recent research on the role of TRF in promoting PDGF-BB during the initial wound-healing stage [59,60]. Hence, we suggest that TRF in this study possibly enhanced PDGF-C to help ischemic tissues revascularise and induce angiogenesis, as well as contributed to the development of normal cerebral vascularization and eventually increased blood flow to reduce the neurovascular dysfunction-associated VaD in rats.

## 4. Materials and Methods

### 4.1. Animals 

Sprague-Dawley (SD) male rats weighing around 180–200 g were used for this study. The SD rats were procured from the AIMST University central animal house. The animal study protocol was approved by the AIMST University Animal Ethics Committee (AUAEC/FOP/2020/12). Rats were housed in the AIMST University central animal house and were fed with a normal pellet diet and fresh water.

### 4.2. Chemicals and Assay Kits

TRF (Gold Tri. E™ 70; Liquid) was received as a gift sample from Sime Darby Oils Nutrition Sdn Bhd. Petaling Jaya, Malaysia. AlCl_3_ (Friendemann Schmidt Chemical, Malaysia), myeloperoxidase activity assay kit (Elabscience Biotechnology Inc., Houston, TX, USA), nitrite assay kit (Sigma-Aldrich, St. Louis, MO, USA), rabbit polyclonal against platelet-derived growth factor C (Abbexa, Cambridge, UK) and rabbit specific HRP_DAB IHC detection kit—micro-polymer v2d, ab236469 (Abcam, Waltham/Boston, MA, USA) were used. All other chemicals used for biochemical estimations were of analytical grade.

### 4.3. Experimental Design

The rats were divided into six groups with 9 rats in each group. The duration of the study was for twenty-eight days. All the rats except normal control (CON) rats were induced with AlCl_3_ (150 mg/kg/bw; *i.p*) dissolved in normal saline [61] for the first seven days. The AlCl_3_-induced rats were further treated with three different doses of TRF dissolved in olive oil (Basso, Italy) as a vehicle [46,62] for the next twenty-one days. Additionally, a standard drug treatment group treated with donepezil (DON) was also included. The animal grouping with their respective treatment is detailed below.

CON: Normal control rats received an equal volume of vehicle.
AlCl_3_: AlCl_3_-induced rats received an equal volume of vehicle.
TRF 30: AlCl_3_-induced rats received TRF 30 mg/kg/bw; *p.o*.
TRF 60: AlCl_3_-induced rats received TRF 60 mg/kg/bw; *p.o*.
TRF 120: AlCl_3_-induced rats received TRF 120 mg/kg/bw; *p.o*.
DON: AlCl_3_-induced rats received DON 1 mg/kg/bw; *p.o.* [63].

### 4.4. Elevated Plus Maze (EPM) Test

The EPM test was performed toward the end of the study to evaluate learning and memory in rats. The EPM apparatus consists of two open arms (50 cm × 10 cm) and two covered arms (50 cm × 10 cm × 40 cm). The arms were connected to a central platform (10 cm × 10 cm) and the maze was elevated to a height of 50 cm from the floor. On day 1 of the EPM test, the rats were individually placed at the end of either of the open arms facing away from the centre and the time taken for the rat to move from the open arm to anyone covered arms was noted as initial transfer latency. Transfer latency is used as a parameter for the estimation of the memory-enhancing property. If the rat did not enter the covered arm within the 90 s, it was gently pushed on the back into one of the covered arms and the transfer latency was recorded as 90 s. Once the rat entered in covered arms it was allowed to explore the arms for at least 20 s. Later, the rats were returned to their home cages and transfer latency was recorded again after 24 h of the first exposure, i.e., on day 2 of the EPM test, and considered as retention transfer latency. The maze was cleaned with a 10% ethanol solution and dried before the next rat was placed on the maze to diminish any odor cues. The transfer latency measured on the first day of the trial served as acquisition (learning) and the second day of the trial served as retention (memory) [64,65,66].

### 4.5. Blood Sample Collection and Brain Homogenate Preparation

Blood was withdrawn from an anaesthetized rat using the retro-orbital puncture method. Plasma and serum were separated from the blood and kept at −80 °C until the time of biochemical estimations. Later, the rats were individually sacrificed, and the brains were isolated. The whole rat brain was then further homogenized in phosphate buffer, and the homogenate was centrifuged at 3000 rpm for 15 min to yield a clear supernatant. The supernatant was collected and kept at −80 °C until the time of biochemical estimations. In addition, two brain samples from each group were taken and preserved into 10% neutral buffered formalin for the immunohistochemistry evaluation.

### 4.6. Estimation of Brain-Thiobarbituric Acid Reactive Substances

The whole brain thiobarbituric acid reactive substances (TBARS) content was estimated following the method of Buege and Aust [67]. Briefly, 0.2 mL of brain homogenate supernatant was mixed with 2 mL of thiobarbituric acid, trichloroacetic acid, and hydrochloric acid reagents in the test tube. Further, all the mixed reagents in the test tube were heated for 10 min in a boiling water bath to develop pink colour chromogen. Later, test tubes were cooled under running tap water and then centrifuged at 5500 rpm for 15 min and the absorbance was recorded spectrophotometrically. The changes in absorbance were recorded using a UV visible spectrophotometer (UV-1800 Shimadzu Spectrophotometer, Shimadzu Corporation, Kyoto, Japan) at 532 nm wavelength. A standard curve was plotted using 0–100 µM of tetramethoxypropane. The brain’s total protein was estimated by following Lowry’s method [68].

### 4.7. Estimation of Serum Nitrite

A nitrite assay kit (Griess Reagent) was used to measure the serum nitrite levels. The assay was performed as per the manufacturer’s (Sigma-Aldrich; Catalog Number: MAK367) instructions. According to the manufacturer’s (Sigma-Aldrich) technical bulletin, ‘The Nitrite assay kit utilises the Griess reagent, a classic protocol for the estimation of nitrite. In the assay, nitrite is reduced to nitrogen oxide using Griess reagent I. Then, nitrogen oxide reacts with Griess reagent II, forming a stable product that can be detected by its absorbance at 540 nm’.

### 4.8. Estimation of Plasma Myeloperoxidase

A myeloperoxidase (MPO) activity assay kit was used to measure the plasma myeloperoxidase levels. The assay was performed as per the manufacturer’s (Elabscience Biotechnology Inc.; Catalog Number: E-BC-K074-S) instructions. The detection principle stated in the manual by the manufacturer (Elabscience Biotechnology Inc.) is ‘myeloperoxidase reduces hydrogen peroxide to a complex. The complex react with o-dianisidine (as hydrogen donor) to produce a yellow product which has a maximum absorption peak at 460 nm. The activity of MPO can be calculated indirectly by measuring the OD value at 460 nm’.

### 4.9. Immunohistochemistry Study for Identification of PDGF-C Expression in Hippocampus

The expression of platelet-derived growth factor-C (PDGF-C) in the rat brain’s hippocampus tissue was determined using an immunohistochemistry (IHC) analysis. A total of 2–3 mm thick coronal sections were cut throughout the dorsal hippocampus. Brain sections were processed in a Thermo tissue processor, according to the manufacturer’s protocol for 16 h, this included fixation, dehydration, clearing, and wax infiltration. Brain tissues sections were embedded in molten paraffin wax (Thermo, Waltham, MA, USA) and cooled out; the formed paraffin wax mould was then trimmed for obtaining 3 um sections using a Leica microtome, and the sections were fished out using Poly-Lysin coated slides (Thermo) for IHC staining. All the prepared slides were left at room temperature overnight for dehydration. The IHC-optimized protocol (Abcam) was followed. The primary antibody (Rabbit Polyclonal against PDGF-C, Abbexa) and secondary antibody (Rabbit specific HRP_DAB IHC Detection Kit—Micro-polymer v2d, ab236469, Abcam) were used. The method was performed according to the manufacturer’s (Abcam) instructions. No primary antibody control slide for each sample was run in parallel with the tested slide. The slides were observed under the microscope to score the intensity of staining.

### 4.10. Statistical Analysis

A two-way analysis of variance (ANOVA) followed by the Bonferroni post-test was applied for EPM data, while a one-way ANOVA followed by Tukey’s multiple comparison test was applied for the biochemical data. The probability value of *p <* 0.05 was considered statistically significant. The data were analysed using GraphPad Prism version 5.03 software.

## 5. Conclusions

The study findings suggest that palm oil TRF can reduce AlCl_3_-induced neurovascular dysfunction-associated VaD in rats perhaps by exerting its antioxidant and neuroprotective effects on NVU. Moreover, our study with TRF suggests that PDGF-C activation using natural antioxidants for facilitating the neovascularisation process in the ischemic tissue could be a new approach in the management of VaD; however, detailed research in this context is recommended.

## Figures and Tables

**Table 1:** Effect of TRF on brain thiobarbituric acid reactive substances.

Treatment	Brain TBARS (nM/mg of Protein)
CON	59.40 ± 2.84
AlCl_3_	83.58 ± 4.80 ^a^
TRF 30	68.71 ± 1.48
TRF 60	53.14 ± 5.21 ^b^
TRF 120	53.84 ± 3.83 ^b^
DON	51.15 ± 1.38 ^b^

Each group (*n* = 6) represents mean ± SEM. ^a^ = *p* < 0.05 vs. CON; ^b^ = *p* < 0.05 vs. AlCl_3_. Abbreviations: AlCl_3_, aluminium chloride; CON, control; DON, donepezil; TBARS, thiobarbituric acid reactive substances; and TRF, tocotrienol-rich fraction.

## Data Availability

The data presented in this study are available on request from the corresponding author.

## References

[1] Tariq S., Barber P.A. (2018). Dementia Risk and Prevention by Targeting Modifiable Vascular Risk Factors. J. Neurochem..

[2] Shabir O., Berwick J., Francis S.E. (2018). Neurovascular Dysfunction in Vascular Dementia, Alzheimer’s and Atherosclerosis. BMC Neurosci..

[3] Wolters F.J., Ikram M.A. (2019). Epidemiology of Vascular Dementia. Arterioscler. Thromb. Vasc. Biol..

[4] Kuang H., Zhou Z.-F., Zhu Y.-G., Wan Z.-K., Yang M.-W., Hong F.-F., Yang S.-L. (2021). Pharmacological Treatment of Vascular Dementia: A Molecular Mechanism Perspective. Aging Dis..

[5] Sashindranath M., Nandurkar H.H. (2021). Endothelial Dysfunction in the Brain. Stroke.

[6] Martins-Filho R.K., Zotin M.C., Rodrigues G., Pontes-Neto O. (2020). Biomarkers Related to Endothelial Dysfunction and Vascular Cognitive Impairment: A Systematic Review. Dement. Geriatr. Cogn. Disord..

[7] Quick S., Moss J., Rajani R.M., Williams A. (2021). A Vessel for Change: Endothelial Dysfunction in Cerebral Small Vessel Disease. Trends Neurosci..

[8] Wang L., Xiong X., Zhang L., Shen J. (2021). Neurovascular Unit: A Critical Role in Ischemic Stroke. CNS Neurosci. Ther..

[9] Patwa J., Flora S.J.S. (2020). Heavy Metal-Induced Cerebral Small Vessel Disease: Insights into Molecular Mechanisms and Possible Reversal Strategies. Int. J. Mol. Sci..

[10] Bai T., Yu S., Feng J. (2022). Advances in the Role of Endothelial Cells in Cerebral Small Vessel Disease. Front. Neurol..

[11] Frisardi V., Solfrizzi V., Kehoe P.G., Imbimbo B.P., Vendemiale G., Capurso A., Panza F. (2011). Aluminium in the Diet, Cognitive Decline and Dementia. Handbook of Behavior, Food and Nutrition.

[12] Maya S., Prakash T., Madhu K.D., Goli D. (2016). Multifaceted Effects of Aluminium in Neurodegenerative Diseases: A Review. Biomed. Pharmacother..

[13] Alfieri A., Srivastava S., Siow R.C.M., Modo M., Fraser P.A., Mann G.E. (2011). Targeting the Nrf2–Keap1 Antioxidant Defence Pathway for Neurovascular Protection in Stroke. J. Physiol..

[14] Burke M.J.C., Nelson L., Slade J.Y., Oakley A.E., Khundakar A.A., Kalaria R.N. (2014). Morphometry of the Hippocampal Microvasculature in Post-Stroke and Age-Related Dementias. Neuropathol. Appl. Neurobiol..

[15] Ismail M., Alsalahi A., Imam M.U., Ooi D.J., Khaza’ai H., Aljaberi M.A., Shamsudin M.N., Idrus Z. (2020). Safety and Neuroprotective Efficacy of Palm Oil and Tocotrienol-Rich Fraction from Palm Oil: A Systematic Review. Nutrients.

[16] Atia A., Alrawaiq N.S., Abdullah A. (2021). Tocotrienols Activate Nrf2 Nuclear Translocation and Increase the Antioxidant- Related Hepatoprotective Mechanism in Mice Liver. Curr. Pharm. Biotechnol..

[17] Sadikan M.Z., Nasir N.A.A., Iezhitsa I., Agarwal R. (2022). Antioxidant and Anti-Apoptotic Effects of Tocotrienol-Rich Fraction against Streptozotocin-Induced Diabetic Retinopathy in Rats. Biomed. Pharmacother..

[18] Ranasinghe R., Mathai M., Zulli A. (2022). Revisiting the Therapeutic Potential of Tocotrienol. BioFactors Oxf. Engl..

[19] Zainal Z., Khaza’ai H., Kutty Radhakrishnan A., Chang S.K. (2022). Therapeutic Potential of Palm Oil Vitamin E-Derived Tocotrienols in Inflammation and Chronic Diseases: Evidence from Preclinical and Clinical Studies. Food Res. Int..

[20] Shaikh S.A., Varatharajan R., Muthuraman A. (2022). Palm Oil Derived Tocotrienol-Rich Fraction Attenuates Vascular Dementia in Type 2 Diabetic Rats. Int. J. Mol. Sci..

[21] Koladiya R.U., Jaggi A.S., Singh N., Sharma B.K. (2009). Beneficial Effects of Donepezil on Vascular Endothelial Dysfunction-Associated Dementia Induced by L-Methionine in Rats. J. Health Sci..

[22] Yokel R.A. (2012). Aluminum in Food–The Nature and Contribution of Food Additives.

[23] Nayak P., Li Y.V., Zhang J.H. (2012). Conjecturable Role of Aluminum in Pathophysiology of Stroke. Metal Ion in Stroke.

[24] Zhao Y., Dang M., Zhang W., Lei Y., Ramesh T., Priya Veeraraghavan V., Hou X. (2020). Neuroprotective Effects of Syringic Acid against Aluminium Chloride Induced Oxidative Stress Mediated Neuroinflammation in Rat Model of Alzheimer’s Disease. J. Funct. Foods.

[25] Ojha P.S., Biradar P.R., Tubachi S., Patil V.S. (2022). Evaluation of Neuroprotective Effects of Canna Indica L against Aluminium Chloride Induced Memory Impairment in Rats. Adv. Tradit. Med..

[26] Durani L.W., Hamezah H.S., Ibrahim N.F., Yanagisawa D., Nasaruddin M.L., Mori M., Azizan K.A., Damanhuri H.A., Makpol S., Wan Ngah W.Z. (2018). Tocotrienol-Rich Fraction of Palm Oil Improves Behavioral Impairments and Regulates Metabolic Pathways in AβPP/PS1 Mice. J. Alzheimers Dis..

[27] Nagapan G., Meng Goh Y., Shameha Abdul Razak I., Nesaretnam K., Ebrahimi M. (2013). The Effects of Prenatal and Early Postnatal Tocotrienol-Rich Fraction Supplementation on Cognitive Function Development in Male Offspring Rats. BMC Neurosci..

[28] Taridi N.M., Yahaya M.F., Teoh S.L., Latiff A.A., Ngah W.Z.W., Das S., Mazlan M. (2011). Tocotrienol Rich Fraction (TRF) Supplementation Protects against Oxidative DNA Damage and Improves Cognitive Functions in Wistar Rats. Clin. Ter..

[29] Bai T., Li M., Liu Y., Qiao Z., Wang Z. (2020). Inhibition of Ferroptosis Alleviates Atherosclerosis through Attenuating Lipid Peroxidation and Endothelial Dysfunction in Mouse Aortic Endothelial Cell. Free Radic. Biol. Med..

[30] Yongxia Z., Jian X., Suyuan H., Aixin N., Lihong Z. (2020). Isolation and Characterization of Ergosterol from Monascus Anka for Anti-Lipid Peroxidation Properties. J. Mycol. Med..

[31] Exley C. (2004). The Pro-Oxidant Activity of Aluminum. Free Radic. Biol. Med..

[32] Abd El-Aziz N.M., Shehata M.G., Alsulami T., Badr A.N., Elbakatoshy M.R., Ali H.S., El-Sohaimy S.A. (2023). Characterization of Orange Peel Extract and Its Potential Protective Effect against Aluminum Chloride-Induced Alzheimer’s Disease. Pharmaceuticals.

[33] Kantar D., Acun A.D., Danışman B. (2022). Effects of Thymoquinone on Scopolamine-Induced Spatial and Echoic Memory Changes through Regulation of Lipid Peroxidation and Cholinergic Impairment. Behav. Brain Res..

[34] Elmorsy E., Elsharkawy E., Alhumaydhi F.A., Salama M. (2021). The Protective Effect of Indian Catechu Methanolic Extract against Aluminum Chloride-Induced Neurotoxicity, A Rodent Model of Alzheimer’s Disease. Heliyon.

[35] Liu L., Liu Y., Zhao J., Xing X., Zhang C., Meng H. (2020). Neuroprotective Effects of D-(-)-Quinic Acid on Aluminum Chloride-Induced Dementia in Rats. Evid. Based Complement. Alternat. Med..

[36] Ahsan H., Ahad A., Iqbal J., Siddiqui W.A. (2014). Pharmacological Potential of Tocotrienols: A Review. Nutr. Metab..

[37] Bardhan J., Chakraborty R., Raychaudhuri U. (2011). The 21st Century Form of Vitamin E--Tocotrienol. Curr. Pharm. Des..

[38] Budin S.B., Othman F., Louis S.R., Bakar M.A., Das S., Mohamed J. (2009). The Effects of Palm Oil Tocotrienol-Rich Fraction Supplementation on Biochemical Parameters, Oxidative Stress and the Vascular Wall of Streptozotocin-Induced Diabetic Rats. Clin. Sao Paulo Braz..

[39] Kuhad A., Chopra K. (2009). Attenuation of Diabetic Nephropathy by Tocotrienol: Involvement of NFkB Signaling Pathway. Life Sci..

[40] Matough F.A., Budin S.B., Hamid Z.A., Abdul-Rahman M., Al-Wahaibi N., Mohammed J. (2014). Tocotrienol-Rich Fraction from Palm Oil Prevents Oxidative Damage in Diabetic Rats. Sultan Qaboos Univ. Med. J..

[41] Wong R.S.Y., Radhakrishnan A.K. (2012). Tocotrienol Research: Past into Present. Nutr. Rev..

[42] Choi Y., Lee J. (2009). Antioxidant and Antiproliferative Properties of a Tocotrienol-Rich Fraction from Grape Seeds. Food Chem..

[43] Virk D., Kumar A., Jaggi A.S., Singh N. (2021). Ameliorative Role of Rolipram, PDE-4 Inhibitor, against Sodium Arsenite–Induced Vascular Dementia in Rats. Environ. Sci. Pollut. Res..

[44] Corzo L., Zas R., Rodríguez S., Fernández-Novoa L., Cacabelos R. (2007). Decreased Levels of Serum Nitric Oxide in Different Forms of Dementia. Neurosci. Lett..

[45] Zhu H.-Y., Hong F.-F., Yang S.-L. (2021). The Roles of Nitric Oxide Synthase/Nitric Oxide Pathway in the Pathology of Vascular Dementia and Related Therapeutic Approaches. Int. J. Mol. Sci..

[46] Norsidah K.-Z., Asmadi A.Y., Azizi A., Faizah O., Kamisah Y. (2013). Palm Tocotrienol-Rich Fraction Improves Vascular Proatherosclerotic Changes in Hyperhomocysteinemic Rats. Evid. Based Complement. Alternat. Med..

[47] Bosche B., Molcanyi M., Noll T., Rej S., Zatschler B., Doeppner T.R., Hescheler J., Müller D.J., Macdonald R.L., Härtel F.V. (2016). A Differential Impact of Lithium on Endothelium-Dependent but Not on Endothelium-Independent Vessel Relaxation. Prog. Neuropsychopharmacol. Biol. Psychiatry.

[48] Haupt M., Zechmeister B., Bosche B., Lieschke S., Zheng X., Zhang L., Venkataramani V., Jin F., Hein K., Weber M.S. (2020). Lithium Enhances Post-Stroke Blood-Brain Barrier Integrity, Activates the MAPK/ERK1/2 Pathway and Alters Immune Cell Migration in Mice. Neuropharmacology.

[49] Karel M.F.A., Roosen M.G.C.H., Tullemans B.M.E., Zhang C.E., Staals J., Cosemans J.M.E.M., Koenen R.R. (2022). Characterization of Cerebral Small Vessel Disease by Neutrophil and Platelet Activation Markers Using Artificial Intelligence. J. Neuroimmunol..

[50] Mohd Nor N.A., Budin S.B., Zainalabidin S., Jalil J., Sapian S., Jubaidi F.F., Mohamad Anuar N.N. (2022). The Role of Polyphenol in Modulating Associated Genes in Diabetes-Induced Vascular Disorders. Int. J. Mol. Sci..

[51] Chianca M., Panichella G., Fabiani I., Giannoni A., Aimo A., Franco A.D., Vergaro G., Grigoratos C., Castiglione V., Cipolla C.M. (2022). Bidirectional Relationship between Cancer and Heart Failure: Insights on Circulating Biomarkers. Front. Cardiovasc. Med..

[52] Zhang X., Sun Y., Zhang Y., Fang F., Liu J., Xia Y., Liu Y. (2022). Cardiac Biomarkers for the Detection and Management of Cancer Therapy-Related Cardiovascular Toxicity. J. Cardiovasc. Dev. Dis..

[53] Cheng H.S., Ton S.H., Tan J.B.L., Abdul Kadir K. (2017). The Ameliorative Effects of a Tocotrienol-Rich Fraction on the AGE-RAGE Axis and Hypertension in High-Fat-Diet-Fed Rats with Metabolic Syndrome. Nutrients.

[54] Saw T.Y., Malik N.A., Lim K.P., Teo C.W.L., Wong E.S.M., Kong S.C., Fong C.W., Petkov J., Yap W.N. (2019). Oral Supplementation of Tocotrienol-Rich Fraction Alleviates Severity of Ulcerative Colitis in Mice. J. Nutr. Sci. Vitaminol..

[55] Tian Y., Zhan Y., Jiang Q., Lu W., Li X. (2021). Expression and Function of PDGF-C in Development and Stem Cells. Open Biol..

[56] Moriya J., Wu X., Zavala-Solorio J., Ross J., Liang X.H., Ferrara N. (2014). Platelet-Derived Growth Factor C Promotes Revascularization in Ischemic Limbs of Diabetic Mice. J. Vasc. Surg..

[57] Grismaldo A., Sobrevia L., Morales L. (2022). Role of Platelet-Derived Growth Factor c on Endothelial Dysfunction in Cardiovascular Diseases. Biochim. Biophys. Acta Gen. Subj..

[58] Grismaldo Rodríguez A., Zamudio Rodríguez J.A., Mendieta C.V., Quijano Gómez S., Sanabria Barrera S., Morales Álvarez L. (2022). Effect of Platelet-Derived Growth Factor C on Mitochondrial Oxidative Stress Induced by High d-Glucose in Human Aortic Endothelial Cells. Pharmaceuticals.

[59] Ting T.M., King J.H., Ho K.L., Lau H.L.N. (2021). Wound Healing Potential of Palm Oil Tocotrienols Rich Fraction. Food Res..

[60] Xu C., Bentinger M., Savu O., Moshfegh A., Sunkari V., Dallner G., Swiezewska E., Catrina S.-B., Brismar K., Tekle M. (2017). Mono-Epoxy-Tocotrienol-α Enhances Wound Healing in Diabetic Mice and Stimulates in Vitro Angiogenesis and Cell Migration. J. Diabetes Complicat..

[61] Liaquat L., Sadir S., Batool Z., Tabassum S., Shahzad S., Afzal A., Haider S. (2019). Acute Aluminum Chloride Toxicity Revisited: Study on DNA Damage and Histopathological, Biochemical and Neurochemical Alterations in Rat Brain. Life Sci..

[62] Lee H., Lim Y. (2018). Tocotrienol-Rich Fraction Supplementation Reduces Hyperglycemia-Induced Skeletal Muscle Damage through Regulation of Insulin Signaling and Oxidative Stress in Type 2 Diabetic Mice. J. Nutr. Biochem..

[63] Chiroma S.M., Hidayat Baharuldin M.T., Mat Taib C.N., Amom Z., Jagadeesan S., Adenan M.I., Mohd Moklas M.A. (2019). Protective Effect of Centella Asiatica against D-Galactose and Aluminium Chloride Induced Rats: Behavioral and Ultrastructural Approaches. Biomed. Pharmacother..

[64] Dhingra D., Parle M., Kulkarni S. (2004). Memory Enhancing Activity of Glycyrrhiza Glabra in Mice. J. Ethnopharmacol..

[65] Rajesh V., Riju T., Venkatesh S., Babu G. (2017). Memory Enhancing Activity of *Lawsonia inermis* Linn. Leaves against Scopolamine Induced Memory Impairment in Swiss Albino Mice. Orient. Pharm. Exp. Med..

[66] Rishitha N., Muthuraman A. (2020). Preventative Effects of Alpha-Naphtho Flavone in Vascular Dementia. Front. Biosci. Elite.

[67] Buege J.A., Aust S.D. (1978). Microsomal Lipid Peroxidation. Methods Enzymol..

[68] Lowry O.H., Rosebrough N.J., Farr A.L., Randall R.J. (1951). Protein Measurement with the Folin Phenol Reagent. J. Biol. Chem..

